# Potential Applications of Social Robots in Robot-Assisted Interventions for Social Anxiety

**DOI:** 10.1007/s12369-021-00851-0

**Published:** 2022-01-25

**Authors:** Samira Rasouli, Garima Gupta, Elizabeth Nilsen, Kerstin Dautenhahn

**Affiliations:** 1 Department of Electrical and Computer Engineering, University of Waterloo, 200 University Avenue West, Waterloo, Ontario N2L 3G1 Canada; 2 Department of Psychology, University of Waterloo, Waterloo, Ontario Canada; 3 Department of Systems Design Engineering, University of Waterloo, Waterloo, Ontario Canada

**Keywords:** Social robots, Social anxiety disorder, Human–robot interaction, Psychological interventions

## Abstract

Social anxiety disorder or social phobia is a condition characterized by debilitating fear and avoidance of different social situations. We provide an overview of social anxiety and evidence-based behavioural and cognitive treatment approaches for this condition. However, treatment avoidance and attrition are high in this clinical population, which calls for innovative approaches, including computer-based interventions, that could minimize barriers to treatment and enhance treatment effectiveness. After reviewing existing assistive technologies for mental health interventions, we provide an overview of how social robots have been used in many clinical interventions. We then propose to integrate social robots in conventional behavioural and cognitive therapies for both children and adults who struggle with social anxiety. We categorize the different therapeutic roles that social robots can potentially play in activities rooted in conventional therapies for social anxiety and oriented towards symptom reduction, social skills development, and improvement in overall quality of life. We discuss possible applications of robots in this context through four scenarios. These scenarios are meant as ‘food for thought’ for the research community which we hope will inspire future research. We discuss risks and concerns for using social robots in clinical practice. This article concludes by highlighting the potential advantages as well as limitations of integrating social robots in conventional interventions to improve accessibility and standard of care as well as outlining future steps in relation to this research direction. Clearly recognizing the need for future empirical work in this area, we propose that social robots may be an effective component in robot-assisted interventions for social anxiety, not replacing, but complementing the work of clinicians. We hope that this article will spark new research, and research collaborations in the highly interdisciplinary field of robot-assisted interventions for social anxiety.

## Introduction

Social anxiety disorder is a condition characterized by a marked and persistent fear of social situations in which feelings of embarrassment or humiliation can occur [1–4]. In social anxiety disorder, the fear or anxiety about experiencing the scrutiny of others is so prominent that people with this condition either completely avoid social situations or endure them with extreme discomfort, despite their desire for social relationships [3].

The onset of social anxiety disorder typically occurs in childhood or early adolescence; 75% of individuals diagnosed with social anxiety disorder have an onset between the ages of 8 and 15 [4]. The onset of social anxiety disorder prior to the age of 11 years increases the risk of disorder persistence in adulthood [5–8]. Behavioural inhibition, which is a consistent tendency to respond to novel situations with fear and withdrawal, predisposes children to the risk of developing maladaptive social anxiety [1, 9–11]. The course of social anxiety disorder can be chronic and lifelong [1–5, 12, 13]. Early diagnosis and intervention are critical for individuals with social anxiety disorder considering the risk of morbidity and disability [2, 14].

### Symptoms of Social Anxiety Disorder

Individuals with social anxiety disorder find many situations of daily life and work difficult and fear- or anxiety-inducing [2, 5, 15, 16]. These include public speaking, being the center of attention, meeting new people, interviewing for employment, conversing with authority, working under others’ observation, entering an already occupied room, attending social events, and even using public restrooms [3, 4, 13]. Individuals with social anxiety disorder often also experience distressing physiological symptoms (e.g., blushing, sweating, trembling, nauseousness, dizziness, muscle tension, rapid heartbeat) during different social situations [13]. Other behavioural manifestations of social anxiety include inadequate assertiveness, rigid body postures, poor eye contact, trembling, speaking with an excessively soft voice, mumbling, stuttering, nail-biting, becoming self-conscious, and withdrawing from social groups or new settings [1, 3, 4]. Young children may express their fear or anxiety in different ways than adults, including crying, freezing, clinging, tantrums, shrinking, or not speaking [4].

### Diagnostic Criteria for Social Anxiety Disorder

**Fig. 1:**
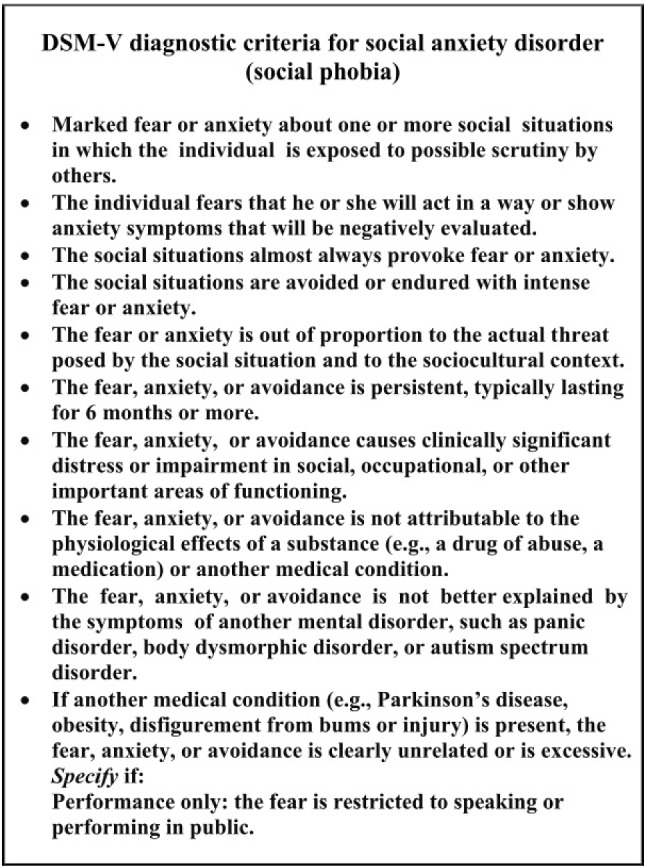
DSM-V diagnostic criteria for social anxiety disorder (social phobia) [4]

**Fig. 2:**
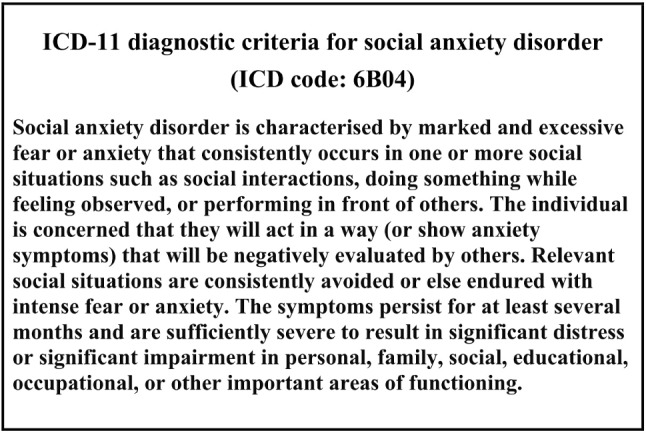
ICD-11 diagnostic criteria for social anxiety disorder (ICD code: 6B04) [17]

An individual can be diagnosed with social anxiety disorder if they meet the diagnostic criteria illustrated in  Figs. 1 and 2.

In addition to the detailed diagnostic criteria, clinicians must consider other features that support diagnosis. Specifically, when assessing children, clinicians must specify that the fear or anxiety occurs in a peer setting and not only during interactions with adults [3, 4].

For all clients, the DSM-V criteria also require clinicians to specify if the fear is ‘generalized’ or ‘performance-only’ [3, 4]. Generalized social anxiety disorder has been defined as a fear or avoidance of a broad range of social situations [3], while the performance-only sub-type of social anxiety disorder is characterized by performance-related fear  [4]. Generalized social anxiety disorder is considered more chronic and severe than the performance-only sub-type.

Social anxiety disorder often co-occurs with major depressive disorder, substance abuse disorders, and other anxiety disorders, and therefore, a differential diagnosis for social anxiety disorder is complicated [1, 3, 4, 13]. In children with high-functioning autism and selective mutism, comorbidity with social anxiety is common [4].

### Prevalence of Social Anxiety Disorder

Social anxiety disorder is one of the most common anxiety disorders, with a lifetime prevalence between 3 to 13% [12, 18–23]. Prevalence rates differ based on various factors, including gender (i.e., higher rates for females than males) [1, 3, 4, 12, 13, 18], ethnic background (i.e., in comparison to individuals of European descent, higher rates have been reported among indigenous communities and lower rates among individuals of Latino, Afro-Caribbean descent, African American, and Asian) [4], marital status (i.e., higher rates among unmarried individuals) and income (i.e., higher rates for individuals from lower-income households) [2, 4].

### Impact of Social Anxiety Disorder

Social anxiety can significantly affect individuals’ overall quality of life [4]. For example, among children and adolescents with social anxiety disorder, lower school performance, refusal to attend school, leaving school prematurely, fewer friends and social isolation have been noted [1, 2, 4, 24, 25]. Despite the reported distress and impairment, only half of all individuals with social anxiety disorder seek treatment, commonly after 15-20 years of experiencing distressing symptoms [3, 4, 12, 26, 27]. Common reasons for treatment delay or avoidance include embarrassment, considering challenges as normative ‘shyness’ or social issue rather than a psychopathology, and anticipatory anxiety (i.e., anxiety from anticipating communication with someone) [4, 12, 20, 25, 28].

### Social Robots and Social Anxiety Disorder

With consideration of the low rates of treatment utilization among individuals with social anxiety disorder due to factors like embarrassment and anticipatory anxiety, it is essential to identify solutions that address and alleviate the barriers to accessing treatments for individuals with social anxiety who could benefit from intervention. Given the technological advancement, one possible avenue to explore is social robots. By definition, ‘social robot’ refers to robots that have been designed specifically to interact and communicate with people in a human-centric and human-compatible manner [29]. “Social robots communicate and coordinate their behaviour with humans through verbal, non-verbal, or affective modalities” [30, p. 3527]. The research finding suggests that interactions with social robots could alleviate anxiety and tension in both persons with and without social anxiety, demonstrating the efficacy of human-robot interactions [31]. Moreover, individuals with social anxiety tend to experience less anticipatory anxiety when they are meeting with a robot than a human  [31].

While certain research projects aim to develop robots that are indistinguishable in appearance and behaviour to humans, the most successfully used social robots across many application areas are robots with a relatively simplistic appearance, but still providing a rich repertoire for verbal and non-verbal interaction and communication with people [29]. For example, such robots can use gaze, facial expressions, body movements, hand gestures, head orientation, control of interpersonal distance (proxemics), and speech to engage people. They can be used in scenarios where they show predictable behaviour in a non-judgmental manner, providing motivating and positive feedback to humans [29]. Robots are programmable machines, they do not show immediate judgmental feedback, as humans would show, unless they are programmed to do so (e.g., a raised eyebrow, changing eye gaze, body posture or prosody). Social robots are typically fully programmable and can thus express a variety of different behaviours, that might be augmented with learning and adaptation mechanisms in order to personalize the robot’s behaviour and allow it to change behaviours over time, for example, during the course of treatment. We posit that these attributes of social robots make them potentially useful as treatment tools in conventional behavioural interventions for social anxiety disorder and that further inquiry in this area is warranted.

In this article, we propose the application of social robots as tools that could complement the support provided by clinicians in the context of intervention for social anxiety disorder. We do so by drawing attention to, and links between, the activities within conventional treatment for social anxiety and the current applications of social robots for interventions in other domains (e.g., work with individuals with Autism Spectrum Disorder (ASD)). The purpose of applying social robots in social anxiety treatment interventions is not only to get people into therapy, but to maximize the effectiveness of therapy through increased engagement and continued support outside the therapy sessions. That is, while the ultimate goal of therapy would be to decrease individuals’ anxiety within human interactions, there may be roles a social robot could adopt to assist in the process of getting to that point.

In the following sections, we briefly discuss evidence-based interventions commonly used for the treatment of social anxiety disorder (Sect. 2), current applications of assistive technologies including social robots, in mental health care and treatment (Sect. 3). Section 4 explains the fundamental characteristics of robots and discusses roles of social robots that have been used in clinical interventions. In Sect. 5, we describe four scenarios as examples of how social robots could be incorporated in conventional behavioural interventions for both children and adults who struggle with social anxiety, as well as associated risks and concerns for using social robots in psychological practice. Section 6 concludes this article.

## Treatments for Social Anxiety Disorder

This section discusses empirically supported interventions that address maladaptive behaviours, cognitions, beliefs, and biases related to social anxiety disorder. We focus on treatments that have demonstrated efficacy, such as cognitive behavioural therapy, exposure therapy, cognitive restructuring, social skills training, cognitive bias modification, and mindfulness-based stress reduction. These treatments tend to be time-limited, goal-oriented and present-focused.

### Cognitive Behavioural Therapy (CBT)

Cognitive models of social anxiety posit that the condition is caused and maintained by expectation and fear of negative evaluation from others [32–34] or revealing perceived flawed aspects of oneself to others [35]. As a result, individuals with social anxiety tend to reduce their anxiety through safety behaviours, such as avoiding others and anxiety-provoking situations. Drawing from models that connect individuals’ cognitions with their emotions and behaviour, CBT is the most extensively studied psychological intervention [36, 37]. It is a time-limited and goal-oriented approach that focuses on the present and teaches patients the cognitive and behavioural competencies required to successfully function in their routine lives [36–39]. The behavioural interventions incorporated in CBT strive to decrease maladaptive behaviours and promote healthier behavioural responses by modifying their precursors and consequences and using consistent practice [37, 39, 40]. The cognitive interventions aim to modify patients’ maladaptive automatic personal interpretations, cognitions, self-statements, or beliefs [37, 39]. Joint effort of the therapist and the patient is an integral component of CBT [36, 37]. This treatment can be delivered both individually and in group sessions [41]. For children, CBT can be delivered in school settings, which offer the benefit of being a natural environment with opportunities for facilitating skill generalization [41–43]. CBT can also include sessions for parents to teach them anxiety management, communication, and problem-solving skills that could promote their child’s progress [36, 41]. For social anxiety disorder, CBT is considered a first-line intervention [3]. CBT incorporates different techniques to address the symptoms of social anxiety disorder. These techniques include, but are not limited to: exposure therapy, applied relaxation, cognitive restructuring, and social skills training [36–38]. In regards to effectiveness, a meta-analysis by Scaini et al. [41] on the effectiveness of CBT for social anxiety disorder in children and adolescents reported that CBT can lead to significant improvement in social anxiety symptoms in both clinical and social settings. Further, a meta-analysis of psychological treatments for social anxiety by Powers et al. [44] found that CBT resulted in better post-treatment outcomes in comparison to being on the wait-list, psychological placebo or pharmacological placebo. Research suggests that gains from CBT are maintained at follow-up [36, 44, 45]. There is also evidence suggesting that, in comparison to pharmacological treatments for social anxiety disorder, the effects of CBT last longer [3, 46, 47]. However, there are several factors that can compromise the effectiveness of CBT, such as poor homework compliance, lower expectancy for improvement, diagnosis of generalized form of the social anxiety disorder, and comorbidity with avoidant personality disorder, mood disorder, substance use disorder, and/or other anxiety disorders [36].

#### Exposure Therapy

This technique is based on the premise that repeated interactions with the fear-inducing situation will gradually initiate the conditioning process of habituation and extinction that is involved in fear reduction [38, 48]. During this intervention, the individual with social anxiety disorder and the therapist work together to create a hierarchy of feared situations (e.g., starting a conversation, speaking in a meeting, presenting in front of a group of people, etc.) that moves from least feared to highly feared situations [25, 36, 38]. The patient then confronts the anxiety-inducing situations through role-plays, out-of-session exercises, imagination, and between-sessions homework assignments [25, 36, 38]. Such confrontation challenges and disconfirms the patients’ unrealistic and maladaptive beliefs about the feared situations and generates a new understanding that competes with the learned fear responses [25, 36, 38, 48]. However, subtle forms of avoidance on the patients’ behalf can compromise the effectiveness of the treatment [36, 38, 48]. Therefore, therapists invest time in identifying safety behaviours and other maladaptive coping strategies that the patients might use during exposure therapy to manage their anxiety [25]. Explicit instruction to maintain focus on the feared situation has also been shown to increase the effectiveness of this intervention [38, 49].

#### Relaxation Training

Relaxation training instructs patients on managing their physiological arousal before or while facing feared social situations by using techniques such as progressive muscle relaxation (PMR) [36, 38, 50]. PMR entails tensing specific muscles for 5–10 s and then releasing the tension [38, 51, 52]. Patients begin with exercising specific, small muscle groups and gradually move to exercise larger muscle groups to accomplish rapid relaxation [38]. By performing this exercise, individuals with social anxiety disorder learn the difference between the sensation of muscle tension and relaxation [38]. This understanding helps them with detecting and releasing muscle tension by remembering the sensations experienced during their relaxed state [38]. Eventually, patients learn cue-controlled relaxation, in which a certain word, often ‘relax,’ is paired with the sensations of relaxation [38]. The cue is then used to initiate rapid relaxation during social interactions [25]. Findings suggest that relaxation training is not effective without an ‘applied’ component to it [36, 38, 53]. In applied relaxation, patients are taught the application of progressive muscle relaxation and cue-controlled relaxation in anxiety-provoking situations, making it a combination of relaxation training and exposure therapy [38, 53].

#### Cognitive Restructuring

The premise of cognitive restructuring is rooted in Aaron Beck’s cognitive model which postulates that biased thoughts, evaluations and beliefs contribute to the development and persistence of psychopathology [54–56]. Hence, in cognitive restructuring, the clinicians and patients collaborate to identify and eliminate inaccurate thoughts experienced during feared situations as well as the beliefs that trigger maladaptive thinking [25, 54]. After identification of inaccurate thoughts, patients evaluate the accuracy of those thoughts by checking data from Socratic questioning or behavioural experiments. Both of these activities are designed to diminish the patient’s negative beliefs about the social situation [38]. Socratic questioning is identified as an essential component of CBT interventions [57] and is defined as “a method of guided discovery in which the therapist asks a series of carefully sequenced questions to help define problems, assist in the identification of thoughts and beliefs, examine the meaning of events, or assess the ramifications of particular thoughts or behaviours” [58, p. 401]. Video and photography feedback may be utilized to correct distorted self-images [25]. Through this process of identification and evaluation, the therapists model disputation of automatic thoughts for the patients [36]. Patients are then encouraged to practise identification and disputation of maladaptive thoughts both during and outside of the session and replace them with more adaptive and balanced thoughts [25, 36].

#### Social Skills Training

According to the skills deficit model of social anxiety disorder, individuals with social anxiety disorder have deficiencies in appropriate social behaviour [36]. These deficiencies elicit negative reactions from others and make social situations anxiety-provoking [25, 36, 38]. As well, findings suggest that individuals with social anxiety disorder tend to underestimate the adequacy of their behavioural performance [38, 59, 60]. Social skills training is often implemented as an intervention for social anxiety disorder. In this intervention, the therapist teaches verbal (e.g., initiating conversations, giving feedback to others) and non-verbal (e.g., maintaining eye contact and an attentive posture) social skills to individuals with social anxiety disorder by modelling the behaviours, engaging in behavioural rehearsal, and providing corrective feedback and positive reinforcement [25, 36, 38]. Commonly, elements of exposure and cognitive restructuring are included to further reduce social anxiety [36, 38]. For example, modelled social behaviours are practised through role-plays during therapy sessions or homework assignments [25].

#### Cognitive Bias Modification

According to the cognitive models of social anxiety disorder, individuals with social anxiety “automatically and selectively attend to socially threatening information (attention bias) and interpret emotionally ambiguous events as threatening (interpretation bias)” [61, p. 2]. Several findings have reported promising results of cognitive bias modification (CBM) as an intervention for social anxiety disorder [61–63]. A growing body of research suggests that social anxiety can be reduced using CBM [61–63]. Findings indicate that CBM might be beneficial as a complementary treatment to traditional psychotherapy [61, 62]. According to the literature, CBM reduces social anxiety by targeting attention and interpretation biases through different experiential tasks [62, 64–67]. CBM targeting attention biases (CBM-A) typically entails a modified dot-probe task [61, 63, 68]. In this dot-probe task, the participant is required to identify, as precisely as possible, the location of a target stimulus that is presented in the place of either a previously presented threatening or positive/neutral stimulus [61, 62, 67, 68]. A quicker response to targets that replace the threatening stimulus indicates an attention bias towards threatening stimuli [61, 62, 68]. In order to modify this bias and teach the participants to attend to the positive/neutral stimulus, practitioners manipulate the frequency with which the target replaces the threatening stimulus [61, 62, 68]. For example, practitioners manipulate the target stimulus to replace the positive/neutral stimuli 80 to 100% of the time [61, 62]. In contrast, CBM targeting interpretation biases (CBM-I) typically uses ambiguous phrases or paragraphs and requires participants to be generative [61, 62]. For example, in the scenario paradigm by Mathews and Mackintosh [69], patients are presented with a short scenario that is ambiguous until the final word, which is a fragmented word that disambiguates in a positive or negative manner [62, 67]. Participants are required to quickly fill in the fragmented word. After solving the word fragment, participants are asked comprehension questions that reinforce the forced positive/negative interpretation [67].

#### Mindfulness-Based Stress Reduction

Rooted in the Buddhist tradition of meditation, mindfulness has been defined as “non-judgemental awareness of the present moment experience” [70, p. 2]. Mindfulness-based stress reduction (MBSR) is one of the most commonly implemented mindfulness-based interventions [71]. MBSR is a highly accessible and inexpensive intervention since it can be delivered in diverse settings and can also be self-taught through the use of books and audiotapes [72]. Typically, MBSR entails different forms of mindfulness practices, such as formal and informal meditation and hatha yoga [71, 73–75]. Formal meditation practices include breath-focused attention, body scans, attending to different sensory modalities, monitoring moment-to-moment experience, and sitting, walking, and eating meditation [73, 76]. In contrast, informal meditation practices entail intentionally shifting attention to present moment awareness and becoming mindful in routine activities [73, 76]. Finally, hatha yoga is a practice of different physical yoga postures [77]. Through these mindfulness practices, patients learn to redirect their attention, thoughts, emotions, and physical sensations, which ultimately assists in anxiety management during stressful situations [70]. Research suggests that MBSR can relieve anxiety, stress, and depression symptoms through altering emotion regulation abilities [73]. Specifically for individuals with social anxiety, learning to intentionally focus attention on external social situations can reduce preoccupation with self-critical cognitions that exacerbate anxiety [72]. Furthermore, practising formal meditation exercises can assist in managing distressing physiological symptoms experienced during feared social situations. Findings suggest that there is a 45% response rate among individuals who complete MBSR [72]. People who complete the treatment have reported increased self-esteem, lower anxiety, and depression as well as a positive impact on their functionality and quality of life [72].

### Adjunct Treatment Approaches

According to Carr et al. [78], people continue to experience dissatisfaction with their lives even after becoming symptom-free through traditional psychological interventions. Hence, introducing positive psychological interventions is useful for enhancing well-being and quality of life for people after the traditional therapies have culminated. The overall goal of these interventions is not to replace traditional clinical psychological interventions, but to complement them [78, 79]. Positive psychology interventions (PPIs) seek to enhance well-being by focusing on subjective experiences, particularly “contentment, and satisfaction (in the past); hope and optimism (for the future); and flow and happiness (in the present)” [80, p. 280]. Common PPIs include activities such as improving the use of character strengths, finding flow, expressing gratitude and optimism, practising kindness and forgiveness, and/or strengthening relationships [78, 81, 82].

Research suggests that under and overuse of specific strengths can result in depressive symptoms [83, 84]. Specifically, among individuals with social anxiety disorder, the under- or overuse of social intelligence, self-regulation, zest, humour, and humility were apparent [83]. Under- or overuse of social intelligence is consistent with the under- or over-awareness of these individuals in social situations [33, 83]. Under-use of self-regulation corresponds to the low perceived emotional control commonly theorized in individuals with social anxiety disorder [83, 85]. Under-use of zest is indicative of the debilitating effects of social anxiety disorder on life and the avoidance behaviours of individuals with social anxiety disorder [83]. Under-use of humour is consistent with the tendency to interpret negative cues more readily than positive ones among individuals with social anxiety disorder [83, 86]. Finally, overuse of humility corresponds to the tendency to avoid both positive and negative external evaluation among individuals with social anxiety disorder [83]. Considering such findings, it would be beneficial to incorporate positive psychological interventions that could facilitate development of and/or improvement in specific character strengths [83].

## Current Assistive Technologies for Mental Health

The field of mental health care has been facing major challenges in connecting people in need of therapeutic services with the available health care providers. There are multiple barriers that limit and/or restrict access to mental health treatments and services. First of the many barriers that limit the accessibility of mental health services is “the dominant model of treatment delivery” itself [87, p. 457]. In this model, treatment is provided in-person either one-to-one or in a group by a highly trained mental health professional within a clinical setting. The reliance of this model on trained professionals has become a problem due to the lack of mental health service providers, especially in remote and rural areas [87–93]. However, in the recent times, telepsychology, which entails the provision of conventional mental health services using telecommunications technology, such as telephones, smartphones, and virtual conferences, has significantly increased and improved access to treatment services [88, 93–97]. Studies have shown that telepsychology is a reliable method of treatment and people both in rural and urban areas are receptive to it [94–96]. Despite the increase in telepsychology, shortage of mental health providers continues to be a problem. The second barrier to accessing mental health services is the financial costs associated with them [98, 99]. For example, in Canada, many community-based, non-physician-provided mental health services are not included in the universal health care system [100]. Therefore, unless families and individuals are receiving treatment through hospitals or other government-funded agencies, they either require third-party insurance for mental health coverage or pay out-of-pocket [101, 102]. A third barrier restricting the accessibility of mental health services is the perceived stigma [98, 99, 103]. Due to the stigmatization of mental illnesses, people in need of professional intervention tend to either completely avoid treatment, delay pursuing treatment, or fail to participate during treatment [104–107]. In recent decades, technological advancements, such as the introduction of telepsychology, Artificial Intelligence (AI)-based smartphone applications, and social robots, have provided a way to address some of these barriers.

Emerging from the concept of telepsychology is mHealth or mobile health, which includes applications on smart devices [96]. Smartphone application developers and mental health researchers have capitalized on the proliferation of smartphone ownership and attachment and have developed applications for assessing and treating mental health conditions such as depression and anxiety. A review by Temkin and Schild [108] outlined 14 smartphone applications for the assessment and treatment of anxiety and related disorders. Assessment applications, including CopeSmart, G-moji, Mobile Mood Diary. Mobiletype, PETE and Sensus, strive to provide self-help to the clients and data to practitioners for better tailoring the more conventional interventions for their clients [108]. Users and practitioners have ranked these applications moderate to high in ease, satisfaction, and providing assistance [108–114]. However, a decline in user engagement over time has also been identified [108, 109, 112]. Treatment applications, such as Anxiety Coach, MindShift, REACH, SmartCAT, StudiCare Stress, and Woebot, provide psychoeducation, relaxation therapy, exposure therapy, and/or cognitive restructuring to reduce anxiety symptoms [108]. Analogous to assessment applications, users ranked treatment applications moderate to high in ease of use, acceptability, and satisfaction [108, 115–127]. However, further research is required to assess the efficacy of the treatment applications when used without conventional interventions [108].

While the aforementioned applications reduce anxiety symptoms, none of them, with one exception, directly target social anxiety. Presently, the Challenger App, which is an unguided, internet-based, self-help application that incorporates the principles of cognitive behavioural therapy (CBT) and gamification, is the only smartphone application that targets social anxiety [128–130]. The application is organized as a game-board and the clients move from one end of the board to the other, while accomplishing self-selected goals, overcoming customized challenges, and receiving psycho-education and community supports [128–130]. The Challenger App also suggests exposure exercises based on the location of the client and the people they might be interacting with [129, 130]. This makes the application particularly useful for implementing exposure therapy [130]. Preliminary studies suggest that participants benefited from using this application [128, 130]. The study found that the number of challenges completed on the application correlated with the treatment outcomes [130].

In comparison to conventional mental health interventions, mHealth-based applications are beneficial innovations due to their high accessibility. The expense associated with the aforementioned applications is minimal compared with traditional psychotherapy [108]. Moreover, smartphone applications allow users to track and share data with health care providers relatively easily, which makes therapy provision more convenient [108]. While mHealth based applications have shown success in promoting therapeutic outcomes, such as reduction of depression and/or anxiety symptoms among users, most of these applications lack evidence-based content and/or input from psychologists and tend to suffer from a decline in user engagement over time [108, 109, 112, 131–134]. Developers have strived to ameliorate this issue through the incorporation of game-based approaches in mHealth applications. The term“applied games” refers to the games that employ “design concepts and qualities from the game world” for serious purposes such as health, education, or social situations [135, p. 101]. Applied games are known to have a considerable impact on improving engagement with mental health interventions and motivating behaviour change because of their *appealing*, *engaging*, and *effectiveness* potentials [136]. Serious games and gamification are examples of applied games. Serious games involve computerized games focusing on serious purposes than mere entertainment [137]. This approach uses gaming as a central and primary medium [138]. In contrast, gamification is a technique that utilizes “game design elements in non-game contexts” to improve the user’s engagement and motivation to adopt specific behaviours [139, p. 1]. An excellent example of gamified applications is the aforementioned Challenger App. Research suggests that participants show better engagement with interventions that are game-like due to their interactive nature [134]. Particularly, games can induce increased engagement and a more sustainable behavioural change because they are intrinsically motivating [140, 141]. Several studies have illustrated the potential of utilizing game-based approaches to increase the impact of online mental health and well-being related interventions [136, 141]. Research shows that game-based approaches can result in social, emotional, and cognitive benefits, such as the development of positive social relationships, a sense of belonging, self-esteem, pride, and strategic abilities, such as problem-solving [142–146].

Even though incorporating gamification is beneficial for mHealth applications, research suggests that the decline in engagement and poor adherence may be a consequence of digital applications’ limited social presence [125]. Social presence, whether of an embodied (physically present) or disembodied (software or virtual) agent, influences the level of engagement and overall success of the social interaction [147]. To further address the challenges associated with engagement and social presence in mHealth applications, the use of socially interactive technologies, such as virtual agents and social robots, to deliver or supplement psychosocial interventions has been explored  [148–151]. Findings suggest that a socially assistive agent, with a relatively higher social presence than that apparent in mHealth applications, can be effective in developing relationships with users, gaining their acceptance and trust, and facilitating therapeutic outcomes [152–154]. It has also been suggested that the combination of the social aspects of virtual agents with e-health interventions makes such interventions much more interactive and engaging [155]. Still, since a virtual agent is not physically present, it induces less psychological response than a fully physically embodied robot does. This issue was studied in a survey of 33 experimental studies, which compared people’s interaction with physical robots and virtual agents [156]. Results from the survey discovered that physically present robots were perceived as more persuasive and positive than virtual agents, and they induced better user performance and more salient behavioural and attitudinal responses [156]. Such findings hint at the potential social robots have in facilitating therapeutic outcomes and consequently, mitigating the current challenges experienced in accessing mental health services.

## Social Robots as Assistive Technology

In this section, we first characterize social robots, followed by a discussion of roles that social robots can play in clinical interventions.

### Fundamental Characteristics of Social Robots

**Fig. 3:**
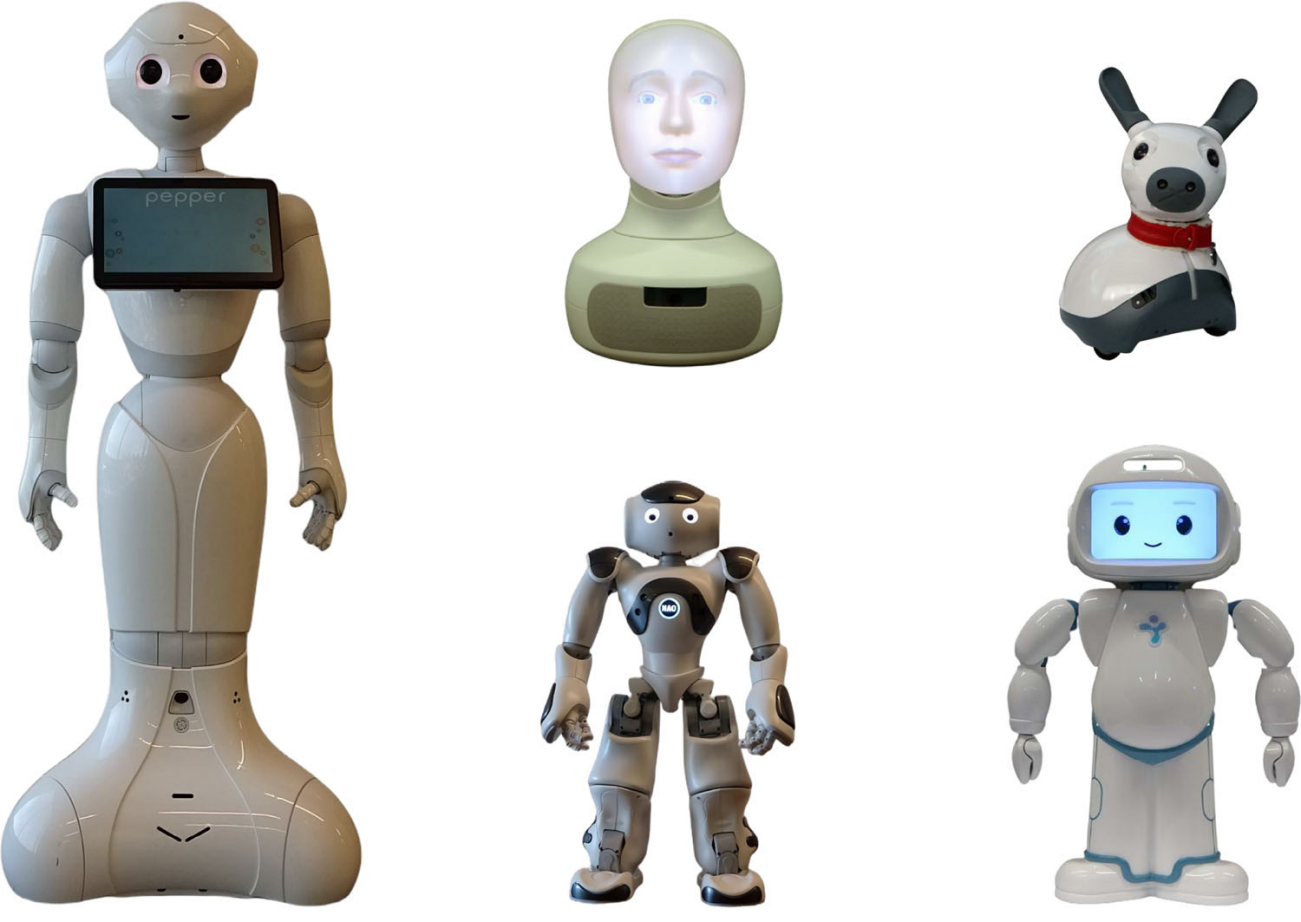
Images of the social robots (from left to right, top to bottom): Pepper (https://www.softbankrobotics.com), Furhat (https://furhatrobotics.com/), Miro (http://consequentialrobotics.com/), NAO (https://www.softbankrobotics.com), and QT Robot (https://luxai.com/)

Social robots are robots that are “designed to interact with people in human-centric terms and to operate in human environments alongside people” [29, p. 1936]. These systems interact with humans by following the behavioural norms and expectations that are defining features of social interaction, such as emotional expressiveness, verbal communication, user engagement, and an appealing physical appearance [157]. Due to their physical appearance, physical availability, and direct interaction in the physical space, interactions with social robots are more natural as well as engaging compared to other forms of interactive technology [158, 159]. The physical appearance of social robots falls on the continuum of human-like (humanoids) to non-humanoids. Humanoids often have expressive faces with oversized, simple features, whereas non-humanoids can be zoomorphic (animal-like), caricatured, or have a purely functional appearance (related to a robot’s tasks) [160]. Examples of social robots are shown in Fig. 3.

Research on the current trends in robot-assisted therapy shows that present interactive robots are either androids (human-like), mascots (human form with cartoon-like features), mechanical robots (human form with visible mechanical features), animal-like robots, or non-humanoid mobile robots [161]. Furthermore, social robots can be either teleoperated (remote-controlled), semi-autonomous, or fully-autonomous. While a remote-controlled robot can adapt to the abilities of the participant and social circumstances, this mode of operation is challenging in the long-term [157, 158] since it requires an operator accompanying the robot. In contrast, an autonomous robot functions without manual operation from a third party and reacts to a current situation based on its perception and analysis of the social circumstances [162]. These robots can either follow pre-programmed scripts or use more complex computational architectures for their decision-making, such as including short-term and long-term memory. Ideally, a social robot’s functionality can also be adaptive and shaped by learning. For example, new skills could be taught to a robot by its owner [160]. While each of these operation modes has its limitations, researchers are aiming to develop autonomous robots that are able to respond in a contingent manner since such robots can reduce the costs associated with traditional therapies as well as the workload of the therapist [163].

### Roles of Social Robots in Clinical Interventions

The use of social robots in delivering mental health care interventions for children with ASD and older adults, especially those with dementia has been widely studied [164, 165]. Studies in these two areas have shown that social robots can effectively engage users and contribute to improvements in their mental health, such as improving their mood, increasing perceived social support, and enhancing the quality of life. Table 1 provides examples of social robots that have been used for therapeutic and other assistive applications in mental health care and well-being.

**Table 1:** Selected examples of robots that have been used for therapeutic or assistive applications

Robot	Picture$^\mathrm{a}$	Description
Kaspar		Kaspar is a humanoid robot developed specifically to support children with ASD. Kaspar can be personalized with different hair and eye colours, clothing, etc [166]
		Example setup for studies into robot-mediated interviews. In this study, typically developing children were either interviewed by a human (left) or a robotic interviewer, i.e., the humanoid robot Kaspar which acted as a mediator, to assist the (hidden) human interviewer (right) [167]. See also applications for children with special needs [168]
IROMEC		IROMEC is a cartoon-like robot used in the European IROMEC project [169]. The robot was designed to support play for children with cognitive, developmental or physical impairments. Other results of the project include the development of different play scenarios for robot-assisted play [170]
Paro		Paro is a pet-like robot in the shape of a baby seal with five different sensors, including tactile, light, audition, temperature, and posture sensors, so that it can perceive its environment and interact with people [171]. Paro has been used extensively in long-term care homes, as shown in the picture on the right [172]
	Credits: AIST, Japan	
Labo-1		Labo-1 is a machine-like robot. The image on the right side shows this robot in the first study in 1998, where children with ASD interacted with an interactive robot. Labo-1 operated autonomously and attracted children with ASD [173, 174]

$^\mathrm{a}$All pictures are used with permission of the authors

Social robots can have different roles to deliver such psychological interventions. According to the review by Diehl et al. [175], there are three potential clinical uses of interactive robots in the particular context of autism therapy. The first clinical application entails eliciting participants’ behaviours for different purposes, such as diagnosis [175]. The second clinical application includes using interactive robots as tools for teaching, modelling, or practising behaviours and skills [175]. The final clinical application of social robots that the authors discuss presents them as tools for providing redirection, reinforcement, and encouragement during triadic social interactions between the child, robot, and the parent/therapist [175].

The available research on the clinical application of social robots points out three major roles for robots in robot-assisted psychotherapy: robots acting as (a) therapists/coaches, (b) mediators, or (c) assistants. As a therapist or coach, the activities of robots are defined and supervised by practitioners to provide a new medium of delivering psychotherapy. In addition to the role of therapists/coaches, social robots can function as mediators that enable or facilitate the treatment progress by mediating interactions between the therapist and client. As a mediator, the robot can also act as a source of motivation and encouragement, which can make the treatment engaging [31, 159]. Finally, as assistants or tools, interactive robots can be used for the elicitation and development of primary social skills, or for assessment/diagnoses [159]. Social robots can play these roles singly or in combination depending on the type of mental health disorders and the associated therapeutic interventions.

Given technological advances in social robots and their growing capabilities, these robots may begin to play an increasing role in enhancing and broadening mental health care interventions and accessibility [87]. Today, interventions employing social robots have been discussed in the context of mental health, such as depression and anxiety disorders. Since it is a relatively new application area of robots, research on social robots for social anxiety disorder is limited. However, the noted benefits of robot-assisted therapy for children with ASD  [157, 161, 175–178] opens up the possibility that social robots could be utilized in other ways, including interventions for mental health challenges, such as social anxiety disorder.

In the following section, we will discuss the possibility of the roles and benefits that social robots can have in delivering interventions for individuals with social anxiety disorder. The potential roles for social robots are outlined with respect to different therapeutic activities that may apply to both adult and child clients, though we recognize that the appropriateness of the roles will differ for the specific population. While the possible roles of robots are not limited to the ones that we are going to describe here, the following sections can provide an overview that can be informative for further research on the influence of interactions with social robots on treatments of social anxiety disorder.

#### Robot-Mediated Interviews

As individuals with social anxiety disorder experience excessive worries in anticipation of social scenarios as well as increased avoidance of social contexts [25], initial treatment sessions may be anxiety-provoking. Moreover, many children or adolescents are not comfortable with novel or unfamiliar situations or people, which may lead to anxiety that interferes with the assessment procedures during the clinical interviews [179]. One possibility for mitigating this challenge could be to incorporate the use of social robots in the interview process.

In recent years, several studies have investigated the possibility of interviewing children with a robotic interviewer [168, 180–183]. These studies focused on applying humanoid robots to facilitate communication and interaction for children, and in particular for children with special needs (e.g., children with ASD) in application areas such as interviewing children about bullying [184], and for evaluating child eyewitness memory [181] as well as in the context of interviews by police or social services [183, 185]. The purpose of these studies is to use robotic interviewers as mediators between professional human interviewers and children to create a comfortable, non-judgemental, and enjoyable setting to engage the children in the interview where the children could more easily express their feelings and experiences [167]. Rather than replacing human interviewers, robotic interviewers are designed to provide human interviewers with a tool that allows them to precisely customize and control the robot’s expressions and behaviour. In addition, an interviewer could have this opportunity to observe participants’ behaviours and non-verbal cues from a third-person perspective [167].

In the field of robot-mediated interviews, various studies have revealed that children’s interaction with robotic interviewers is extremely similar to their interaction with human interviewers, regardless of the difficulty of questions [167, 180]. Results showed that children provided both the human interviewer and the robotic interviewer with equivalent content, i.e., similar amounts and types of information [167, 168, 182]. These results suggest that children responded to robotic interviewers and were engaged within the interviews. According to the results of qualitative analysis, some children with special needs may have been more interested in the robotic interviewers than the human interviewers [168]. The results of a study by Bethel et al. [184] showed that children reported occurrences of bullying significantly more when interviewed by a robot than by a human interviewer. Furthermore, feedback from potential real-world users such as educational psychologists and health care specialists indicated that robot-mediated interviews could be beneficial in real-world applications if the system was sufficiently flexible [183, 185]. They also stated that such systems could have particularly promising applications for children with conditions such as ASD or anxiety that cause communication difficulties and make it hard for these children to communicate with adults in general, and unfamiliar adults in particular. In these cases, a robot as an interviewer could be a useful tool in hands of mental health workers or clinical psychologists for providing counselling services [183].

Previous studies suggest that individuals with social anxiety who experience excessive fear of negative evaluations, which is one of the main symptoms of social anxiety, tend to evaluate interactions with a robot positively, with less tension and stress [31, 186]. Moreover, the anxiety symptoms experienced during interaction might be more manageable for the person [31]. Thus, it may be beneficial for individuals and specifically children or adolescents with social anxiety to initially participate in robot-mediated counselling sessions to increase comfort and engagement. If individuals with social anxiety find it more comfortable to communicate with a robotic interviewer than with a human interviewer in counselling sessions, interaction with a robot-mediated interviewing system may be advantageous, particularly within earlier sessions. While a different population group, it has been found that individuals with ASD, based on numerous studies showing that they generally respond very positively to robots [157, 176, 187–189].

#### Social Robots as Screening/Diagnostic Agents

Behavioural assessment can be used to observe and characterize social behaviours directly [190, 191]. Behavioural assessment can be applied in conjunction with traditional diagnostic interviews and self-report inventories for a comprehensive assessment of patients’ social difficulties [190]. Based on studies, behavioural assessment tests have discriminative validity in distinguishing between young people with and without social anxiety disorder [16, 191, 192]. This approach can also identify social behavioural differences between children and adolescents who are diagnosed with social anxiety disorder [16]. In a group context, this method may use role-playing as a behavioural observation strategy, during which an individual with social anxiety disorder and a peer may perform role-playing tasks using scripted scenarios. It is also possible to include unstructured peer interaction tasks [193]. However, finding appropriate peers in terms of age, availability for needed training, and interaction is a challenge for conducting valid behavioural assessment tests [193].

Social robots have the potential to assist therapists in studying behaviours associated with social anxiety. As discussed in Sect. 1.1, individuals with social anxiety may exhibit behavioural symptoms such as avoiding eye contact, rigid body postures, and inappropriate speaking voice due to the tension and fear caused by social anxiety. Social robots are capable of detecting human social cues and behaving accordingly. Besides, advanced sensors used in the design of social robots could be useful to capture and quantify a wide range of verbal and non-verbal behaviours during clinical sessions. For example, in comparison to typical clinical evaluations of eye contact, gaze direction can be recorded and analyzed in greater details including mutual or averted gaze, number and duration of fixations and gaze points [194]. Furthermore, machine learning algorithms can be employed to recognize human gaze patterns and behaviours such as head movements and facial expressions associated with anxiety and depression [195, 196]. This quantitative information could be made available to the therapist in real-time to enhance diagnosis and initial and ongoing assessment as treatment progresses.

In the context of ASD, it is proposed that social robots could use two methods of passive observation and structured interactions for quantitative measurements of social responses [177]. Robots can record information on social responses through passive observation of the therapist and the client during standard clinical evaluations without directly engaging in interaction. Whereas in structured interactions, the robot can directly engage in interaction to elicit a specific social behaviour or response. The collected information from the passive and interactive methods could provide reliable quantitative measurements, to not only compare different individuals in a standardized manner but also track the progress of each individual over time [177]. With respect to structuring behavioural assessments, social robots could be used to play the role of a peer. The robot could be programmed to demonstrate a wide range of anthropomorphic characteristics, behavioural repertoires, as well as sensory and interactive capabilities. Moreover, social robots could be programmed for various scenarios to focus on specific or single behaviours. In this context, social robots might be used as clinical decision support tools to track various social anxiety indicators, including verbal and nonverbal behaviours, as well as physical and cognitive symptoms. This information could be used by therapists to form case conceptualizations and track progress across sessions.

#### Robot-Assisted Therapy

The benefits of applying robots in therapeutic interventions for children with ASD and individuals with dementia reflect a promising development in robot-assisted therapy [164, 176]. Robot-assisted therapy can be applied to improve patients’ cognitive, social, emotional, and physical functions. In the context of social anxiety, social robots could be incorporated in traditional therapies for social anxiety disorder, such as cognitive-behavioural therapy (CBT), and like any other form of treatment, they could be tailored to suit each client’s needs. Such incorporation of interactive robots could function as a “stepping stone” for individuals with severe social anxiety. For example, they could practice social skills and behaviour before applying them in real-life interactions. Therapeutic sessions could involve facilitating the learning of various social and communication skills. Meanwhile, individuals can practise these skills based on their capabilities to be prepared for real-life scenarios. Confronting feared social situations without the pressures of interacting with another person can add value to existing forms of treatment, such as exposure. Due to this added layer, the anxiety symptoms experienced during exposure might be more manageable, which could potentially encourage the individual to continue treatment and eventually confront social situations, including other people, which is the ultimate goal. Besides, the inclusion of robots in the therapeutic context may introduce the element of comfort and fun into the therapeutic session, which could contribute to emotional support and stress relief during stressful situations within the therapeutic process. The treatment sessions would become more friendly and less threatening, and clients may feel more calm and relaxed when a robot participates in treatment sessions.

Integrating social robots in therapeutic environments could provide support for therapists as well. In a study [197], therapists of an autism therapy center participated in ten focus group sessions to discuss possible roles of robots within the therapy environment that would be beneficial to therapists. Suggested roles are including (a) *“As a helper in critical/dangerous situations”*, for example, a robot could be utilized to distract or ask for help from another therapist, (b) *“As a record keeper and reporting device”* to facilitate preparing reports and help with enhancing therapeutic feedback via activities such as recognizing and analyzing client’s actions, monitoring and evaluating the progress, (c) *“As an emotional support/mirror”*, a robot could be used to suggest a break, distract to lighten the mood, or mirror a client’s emotions, (d) *“As a team player”* to alleviate therapist’s workload and mental load (e.g., by distracting and entertaining a client during break activities, repeating therapists instructions, reminding and managing of schedule). While this article [197] focused on the needs of autism therapists, some of these functionalities and roles can be extended to other areas of psychotherapy. This being said, similar studies need to be conducted to develop a better understanding of therapists’ needs and expectations about the robots in other psychotherapy domains.

#### Social Robots as Interactive Social Companions

Individuals with social phobia report fewer friends and increased difficulty managing their friendships and relationships [198]. Social robots are commonly employed in the role of companions to improve the health and psychological well-being of their users. In some situations, animal-like robots are used, which are designed to function in a way that resembles trained therapy animals. Different studies have investigated the role of animals in mental health care and their advantages in the treatment of mental illness [199]. It has been shown that animal-assisted therapy has a beneficial impact on human anxiety levels and reducing anxiety symptoms [200]. Three separate studies have noted that the presence of a therapy dog in university or college settings significantly decreased students’ self-reported anxiety levels [201–203]. Another study identified the efficacy of equine-assisted therapy and cognitive-behavioural health strategies in alleviating social anxiety symptoms in young women [204]. The completely non-judgmental acceptance of individuals by animals is a characteristic that may be particularly appealing to those who have social anxiety or experience social rejection [205].

Social robots may offer benefits of animal-assisted therapy without the practical concerns and challenges involved in working with live animals such as animal welfare, allergies to animals, safety, and risk of contamination or infection transmission [206]. Currently, numerous pet-like robots are available with the aim of simulating the effects of therapy animals. Several studies have emphasized the positive impact of interacting with robotic pets on mental health, such as enhanced socialization and mood, as well as reduced depression, stress, and anxiety [149, 207–209]. Many of these studies primarily focused on older adults and persons with dementia.

Aibo and Paro are examples of extensively used pet-like companion robots. Aibo is a non-verbal, dog-shaped robot with various sensors through which it can respond to speech, touch, sight, sound, and it can express emotional responses. In a Randomized Controlled Trial (RCT), 38 participants were randomized to interact with the Aibo robot, a real dog, or no object as a control group. At week seven, participants in the dog and Aibo groups reported significantly lower feelings of loneliness than those in the control group, but no significant differences in loneliness or attachment were observed between the dog and Aibo groups [210]. This study supported the effectiveness of using an animal-like robot for pet-therapy. Paro is another most used interactive companion robot in the shape of a baby seal with five different sensors, including posture sensors, light audition, temperature, and surface tactile. Using these sensors, Paro is able to recognize its environment and people. It is shown that interacting with Paro facilitated and increased positive social interactions among residents of a nursing home [209] and also among users and their caregivers [211]. In laboratory studies, children who interacted with the Paro robot indicated a reduction in stress levels and a promotion in positive mood [212].

These studies, along with many others, suggest that social companion robots may provide an engaging and interactive tool for individuals with social anxiety through helping with the management of anxiety levels and associated challenges through providing in-home resources and services. Interactive social robots could also be used to teach essential social skills to younger people with social anxiety who might not be readily interested in the treatment. The robots’ adaptability and ability to perform repeated tasks would also make social robots suitable for teaching, modelling, and/or practising new social skills [161]. At the University of Washington, researchers are working with an emotional and social robot called Emobie that provides in-home companionship to children with anxiety who might not have access to professional therapists [213]. Emobie teaches children coping skills through a storytelling scenario during which this robot listens to the children and responds to them using expressive facial expressions, arm movements, sounds, and colours displayed on a screen located in the abdominal region of the robot. Emobie is designed to assist in the improvement of communication between children and their parents, and/or therapists by communicating the child’s emotions [213].

Furthermore, social companion robots could improve their users’ mental health by engaging them in pleasurable activities. Social anxiety has been shown to be associated with depressive symptoms, and a synergic relationship between scores of social anxiety and depressive symptoms has been observed [214, 215]. Engaging in pleasurable activities and increasing access to positive reinforcers are shown to be effective practices for improving depression and anxiety [216, 217]. Social robots could learn and engage socially and emotionally with users over various sensory inputs such as audio, visual, and tactile. Meanwhile, they may offer education, skills training, mindfulness/relaxation practices, and health-tracking to users, according to their preferences and physical/mental conditions. Social robots could also be used as assistive and supportive companions in delivering interventions designed to enhance well-being, particularly positive psychology interventions, which focus on helping people flourish with positive emotions and personal strengths and skills [218]. It is believed that experience of pleasure, engagement, and meaning, emphasized in positive psychology, in an individual’s life is associated with relieving negatives states and coping with mental health issues, including anxiety [219, 220]. A systematic review into the influence of positive emotions in depression treatment revealed that the different strategies of positive psychology, focused on positive emotions, can contribute to significant improvement in depression signs and symptoms. This study also highlighted the relationship between humour and positive emotions [221]. In the field of social robotics, researchers have investigated the efficacy of providing positive psychology interventions via a robot. In a pre-post-study, a social robot companion, called Jibo, was used to deliver positive psychological interventions, such as character strengths, and gratitude, and build rapport with 35 college students living in on-campus dormitories. Deployed in participants’ rooms, the robot guided the participants on the positive psychological intervention and a pictorial survey. After seven sessions of interacting with the robot, participants showed statistically significant improvement in their psychological well-being, mood, and readiness to change health-related behaviours for further improved well-being. During the post-study interview, other than expressing some privacy concerns, students expressed appreciation for the robot’s companionship, and desire to talk and communicate with the robot [222].

#### Social Robots as Peer/Interactive Playmates

Interactive robotic playmates have been extensively used in therapy applications most often for children with ASD [223, 224] and in learning environments to support children’s language learning  [158, 225, 226]. In the field of autism therapy, social robots are usually involved in fun and engaging activities such as games in which social robots often play the role of therapeutic toys or play partners to encourage basic communication and aid children in practising social interaction skills [173, 206, 227].

Social robots’ physical presence and properties such as communication via natural language, gestures, facial expressions, eye contact can create a rich interactive environment where a user could practise specific skills with the robot. Furthermore, repeatability and predictability of social robots’ behaviour are great advantages of robots over humans. Robots provide an opportunity to practise and explore social skills without fear of the complexity and multi-modality of human-human interaction. Social robots as a “therapeutic teaching device” can participate in enjoyable dyadic human-robot play activities to teach the necessary social skills and engage users in therapeutically relevant interactions [224, p. 447]. In this type of interaction, the robot will direct all its attention to a single person. Besides, it is possible to design play activities that are personalized to a person’s specific needs, preferences, and capabilities. For example, the level of teaching social skills could be adjusted incrementally based on each user’s progress.

Individuals with severe social anxiety may benefit from practising communication and social skills via interacting with social robots as a peer or interactive playmate. These interactions may help individuals with social anxiety become familiarized and comfortable with basic styles of interaction, such that they are more willing and confident to engage in real-life interaction with humans.

#### Social Robots as Social Mediators

Individuals with social anxiety tend to find it hard to participate in social situations due to communication burdens and the fear or concerns associated with the scrutiny of others. A social robot as a social mediator is a tool to encourage and facilitate social interaction between two or more persons or between the person and the therapist (e.g., if a highly anxious child is unwilling to interact with a therapist directly).

Social robots have been successfully used as mediators for children with ASD for many therapeutically relevant areas, such as touch, joint attention, eye contact, turn-taking and sharing, robot behaviour imitation, and cooperation [161, 170, 173, 176, 228–230]. Based on the literature, social robots can facilitate social exchanges between a child with ASD and a partner by incentivizing communication, eliciting and reinforcing social behaviour as well as providing feedback and encouragement [231]. In an observational study, Werry et al. [173] showed that scenarios with a robot as a mediator and pairs of children can create a particularly fascinating social context for observing various social and non-social interaction patterns. Analyzing these behavioural patterns can help to identify specific problems as well as the abilities of children with ASD in social interactions. Giannopulu and Pradel [232] explored the role of a robot as a mediator between a child with autism and a therapist. In this study, the child used the robot as a tool to convey positive emotions to the therapist.

Robins et al. [230] conducted three case studies in which interacting with a minimally expressive robot, Kaspar, encouraged low-functioning autistic children to interact with other children to break their isolation and generalize this behaviour to co-present others. Kaspar is a humanoid robot developed to support children with ASD [166, 233]. This robot has shown the potential to be incorporated into current educational and therapeutic interventions for children with ASD [234]. Recent studies in robot-assisted therapy have indicated how using Kaspar, as a mediator, can help children with ASD to develop and improve skills, such as visual perspective-taking [235]. It is also shown that play sessions with Kaspar have a positive influence on some children’s behaviours in particular areas including communication and interaction, imitation, prompted speech, focus, and attention [236].

In comparison to other social robots’ roles, such as companions or coaches, social robots as mediators will mainly focus on enhancing human-human interaction to support social interactions between people. In the research area of robot-assisted therapy for children with ASD, social robots have been shown to be helpful tools for motivating and reinforcing social engagement of children [157, 176, 189]. This approach could potentially be generalized to other health conditions such as depression or anxiety where individuals’ phobias or social behaviour deficits will affect their social interaction [5].

Designing social robots as social mediators in the particular domain of social anxiety requires the careful study of how social robots can motivate and support individuals with social anxiety to participate in social interactions with one or more partners. For example, social robots could support socially anxious children by prompting and facilitating conversation between youth interacting, moderating social interactions, providing positive reinforcement to increase confidence, promoting conversation inclusiveness and participants’ engagement (e.g., [237–239]) and offering social feedback (e.g., [240]) to develop and practise social skills.

The ultimate goal of interactions with a social mediator robot is to enable individuals with social anxiety to transfer and generalize their learned social skills to their daily social interactions with familiar or unfamiliar people. Thus, as a final stage of such interventions, after a participant has shown sufficient improved interaction skills, the robot will in fact no longer be needed, and individuals would instead continue practising learned social skills with a human partner to further enhance and promote communication and social interaction skills. However, it should be noted that the possibility of transferring and generalizing learned skills from human-robot interaction to human-human interaction is an unsolved issue and requires substantial further research. In a recent study, an autonomous social robot, Jibo, had been used for a month to provide a home-based intervention to improve the social skills of 12 children with ASD, who were between 6 and 12 years old [241]. The results of the study provide evidence that it may be possible to transfer learned social skills beyond robot-mediated interactions to human-human interactions; however, long-term preservation of improved skills still remains a challenge [241].

#### Social Robots as Coach or Instructors

There is a growing interest in developing social robots acting as an instructor or a coach to monitor and engage users in a number of therapeutic or non-therapeutic tasks in a highly personalized way to improve their social, physical, or cognitive well-being. For example, Kidd and Breazeal [242] developed Autom as a behaviour change coach to facilitate sustained engagement in a diet and exercise program through tracking each participant’s weight-loss and providing personalized feedback. In a between-subjects and longitudinal study of six weeks, Autom was compared to a standalone computer and a paper log. After six weeks, participants who took part in the weight-loss program with Autom continued participating in this program for significantly more days than participants in the two other conditions. Besides, a significantly closer working alliance with the robot was reported. In another study, Fasola and Matarić [154] designed and developed Bandit, a robotic coach system, to engage older users in physical exercises. In this study, the robotic coach methodology has been developed based on psychology research on users’ intrinsic motivation. Besides, the researchers compared the physically present Bandit robot to its virtual version to explore the role of physical embodiment. The results showed that physical embodiment had a positive effect on participants’ evaluations of the robot and the interaction. Enjoyableness, social attraction, helpfulness, social presence, and companionship were identified as factors that influence participants’ preferences for the physically embodied robot coach over the virtual coach.

In the context of social anxiety, social robots as coaches or instructors could be incorporated into the treatment of social anxiety for social skills training, cognitive restructuring, mindfulness practices, and relaxation training. A social robot as a social skills training coach could be used to address verbal and non-verbal behavioural deficiencies that may emerge in social situations. This intervention can entail teaching verbal social skills (e.g., how to initiate a conversation and give others positive feedback during a conversation, verbal qualities such as volume, tone, and rate, etc.) and non-verbal social skills (e.g., eye contact, facial expression, body postures, use of gesture, etc.) via behavioural rehearsal, corrective feedback, and positive reinforcement. For instance, public speaking anxiety is one of the prevalent social phobias. A great deal of stress and frustration resulting from excessive public speaking anxiety can negatively affect speech performance and lead individuals to further avoid situations that require public speaking. Good public speaking skills are essential in terms of educational achievement and career success. Those skills can be learned through appropriate training and practice. To facilitate public speaking training, various interactive technologies such as mobile applications, intelligent interfaces, and virtual agents have been developed to promote presenters’ learning experience by providing them with automated feedback on their verbal and non-verbal behaviours [243–249].

The RoboCOP is a robotic coach for public speaking [250] that uses an anthropomorphic robot head called the Furhat robot [251]. This system aims at simulating an interactive rehearsal of real-life presentations to mitigate presenters’ public speaking anxiety. This robot can perform the role of an audience as well as a coach and can provide verbal feedback on presenters’ speech quality (e.g., speaking rate, pitch variety, and filler rate), content coverage, and eye contact. The design of the feedback strategies was based on an exploratory study with eight professors from different disciplines who had experience in guiding students on their lectures or were teaching public speaking classes. RoboCOP also offers high-level advice on other aspects such as presentation goal, audience benefits, talk organization, and how to present a strong introduction and close a speech. To facilitate the rehearsal, the authors designed a topic-based note authoring interface that allows presenters to prepare and segment their speaking notes into a series of key topics for each slide. During rehearsal, RoboCOP tracks the presenter’s speech using automatic speech recognition to provide feedback on content coverage based on the covered key topics on each slide. The Rehearsal procedure consists of two spoken rehearsal modes: *Slide Walkthrough mode* and *Dry Run mode*. During the Slide Walkthrough mode, the presenter practises verbalizing slides, and the robotic coach, as an audience, provides preliminary feedback after each slide. Then, the presenter performs a complete presentation in Dry Run mode, in which the summative feedback will be provided upon completion of the rehearsal to avoid interrupting the presentation flow. To evaluate the impacts of verbal feedback and physical embodiment of RoboCOP on the presenter’s experience, 12 participants with different levels of presentation experience were recruited. Three feedback modalities, including RoboCOP coaching feedback, graph-based visual feedback as well as voice feedback were compared in a within-subject study. Results indicated that practising the presentation with RoboCOP has significantly improved the presenters’ rehearsal experience in comparison with two other feedback modalities. In a second evaluation study, a panel of 12 judges, including students, researchers, and professors with different experience levels in presentation, rated the presenter’s rehearsal with and without the RoboCOP. Results showed that RoboCOP-assisted presentations significantly benefited from an interactive, motivating, and natural rehearsal environment, which resulted in significant enhancements in the quality of presentations. Besides, participants who practised the presentation with RoboCOP expressed great satisfaction and desire to use RoboCOP for their future presentation rehearsals.

RoboCOP is an example of applying robots to social skills training to mitigate public speaking anxiety. Numerous participants with a fear of public speech expressed that they were more comfortable practising presentations with RoboCOP compared to human audiences. They also reported that the presence of the robot helped them practise maintaining eye contact during the presentation rather than looking at their notes [250]. It can be argued that the robot’s physical embodiment and its eye gaze tracking capability are the advantages of this platform over other media for public speaking training. This platform could also be developed to deliver feedback on a presenter’s body language and facial expressions. To improve the effectiveness of the training process, future systems need to include mechanisms that could dynamically set realistic objectives based on each presenter’s characteristics, performances, and anxiety level. For example, the coach’s frequency and timing of feedback and suggestions could adversely affect certain participants’ confidence levels and increase their anxiety, particularly when they do not show any noticeable improvement. Thus, further research is required to adapt the behaviour of the robotic coach dependent on presenters’ level of abilities and anxiety to provide more personalized feedback and suggestions as well as a non-threatening experience for different users.

In addition, social robots can present a novel approach to address individuals’ social anxiety by delivering cognitive restructuring training and practice. As noted earlier, one aspect of CBT is identifying and modifying the maladaptive thoughts associated with anxiety (e.g., negative evaluation or scrutiny from others). As a non-judgmental, stress-reducing, and encouraging agent [252, 253], social robots could be advantageous for applications in cognitive restructuring. As a first step of implementing cognitive restructuring training, the robot could help users to identify the negative or irrational self-related thoughts that produce distress before, during, or after the specific social situation, identify corresponding emotional states, and assist individuals in replacing those inaccurate thoughts with more positive and rational statements through providing a series of questions (e.g., what evidence is there? what would you say to a friend? etc.).

In a recent study, a conversational agent, Amazon Alexa, has been used to address public speaking anxiety through cognitive reconstruction exercises. Alexa, as a coach, interacted with participants through structured conversation scripts and instructed them to imagine themselves presenting a speech to identify the participants’ negative self-focused statements during public speaking. Next, Alexa taught participants to substitute their negative thoughts, such as *“What I say will probably sound stupid”* with positive and adaptive coping statements such as *“There’s nothing to lose. It’s worth a try”*. The results of the study on 53 college students, who had moderate to an intense fear of public speaking, revealed that the interaction with Alexa helped to alleviate pre-speech state anxiety. In addition, the sociability of Alexa (e.g., self-introduction, showing empathy, and using conversational fillers) increased students’ satisfaction and willingness for future engagement. In this study, participants also discussed Alexa’s weaknesses as a public speaking coach, including machine-like interaction and lack of anthropomorphization, lack of flexibility in response time for different participants, lack of personalized advice, and feedback [254].

A social robot as a coach or instructor can also offer an innovative approach for mindfulness practices (e.g., instructing meditation and hatha yoga exercise) and relaxation training for the management of the physiological arousals such as rapid heart rate, sweating, blushing, and trembling that often accompany anxiety. Robots could teach individuals how to relax in anxiety-provoking situations starting with training progressive muscle relaxation. In this intervention for social phobia, users learn how to attend to and control physiological arousals before or during exposure to feared social situations [36, 38].

Incorporating the social robot as a coach or instructor into the treatment of social anxiety could facilitate coaching in real-life settings and allow individuals with social anxiety to acquire and practise cognitive and behavioural skills for different social situations in which they may experience debilitating fear or anxiety. Social robots can create interactive learning environments for users to practise specific skills and receive timely feedback. By supporting experiential learning, social robots have the potential to improve the effect of coaching interventions and reduce associated costs. Furthermore, these robots can be designed for the means of facilitating a variety of therapeutically relevant functions both inside and outside of therapy sessions with clinicians [206]. Within a therapy session, social robots can provide direct guidance to and monitoring of clients participating in relevant therapy activities defined by a human provider. Outside of the therapy sessions, these robots can be used to engage and encourage users to perform and practise therapy relevant activities and homework assignments [206]. In this setting, robotic coaches are platforms that could assist users in adhering to treatment by providing education and real-time corrective or motivational feedback, as well as monitoring the treatment progress.

**Table 2:** Scenario 1: game of social skills

Target group	Children and adolescents
Role of the robot	Peer or friendly playmate in one-to-one sessions. In group sessions, the robot would presume the role of a mediator and provide positive reinforcement to the group
Role of the participant	Learner
	In individual sessions with a robot and a clinician, the youth could learn different social skills and the most effective way to implement them. In this psychoeducation stage, the social robot could model the social skills for the participant. After psychoeducation, the robot and the participant would engage in a one-to-one game with rules. In the game, the robot and the participant would use a dice to arrive at different locations on a game board that corresponds to particular social skills that the participant needs practice in. Upon arriving at a location on the game board, the party who rolled the dice would perform the social skill specified for that location. Based on the inherent difficulty of each social skill, a score would be allotted to the skill. Higher scores would be allotted for successfully performing more complex social skills. The party with the most points wins the game
Place/setting	Home, school or clinic
Level of difficulty/variations	Within a group context, youth would engage in the same board game with peers in a robot-mediated group intervention session. In this context, the robot is no longer an individual playmate, but instead could be a mediator for the group, supporting all members in practising and demonstrating successful interaction skills. This variation would be useful for participants who have completed the individual sessions with the robot as a playmate
Potential benefits	With the incorporation of a social robot, youth may be able to learn different social skills in an engaging manner during psychoeducation. Social robots interact in simple and predictable manners which offer an opportunity to practise social skills without fear of the complexity of human-human interaction and perceived scrutiny of others. With repeated practice, children may develop increased confidence such that they are more willing to engage in social interactions in real life

**Table 3:** Scenario 2: simulating social situations

Target group	Children, adolescents and adults
Role of the robot	Coach or instructor that would rehearse with a participant simulated versions of feared social situations
Role of the participant	Performer
	This activity could involve practising complex social skills in simulated social situations. Before the beginning of this activity, participants would be asked to list feared situations in a hierarchy, beginning with least feared to highly difficult. Then the participant and the robot would engage in a simulation of a feared social situation. The social robot could be programmed to demonstrate specific verbal and nonverbal behaviours associated with the role. For example, if the simulated social situation is a job interview, the robot would presume the role of the interviewer. The robot could also provide verbal positive reinforcement to encourage participants and build their confidence
Place/setting	Home, school or clinic
Level of difficulty/variations	Participants would start with contexts seen as easier on their list and move upwards to more-difficult contexts once they demonstrate mastery and report comfort. The ultimate aim would be for participants to engage in these scenarios in real life. That is, the work with the social robot would provide lower levels of hierarchy and as the participant masters these, real-life scenarios would be attempted
Potential benefits	Through this activity, participants could learn to tolerate and see themselves engaging successfully within various situations within a controlled setting. They could try out and experience themselves demonstrating social and communicative behaviours within typically anxiety-provoking social situations with the social robot as many times as needed in order to become comfortable and confident, such that they can then practise the learned behaviours in the ‘real world’. For younger participants, the social robot could provide a progress report at the end of each session, which the youth could view later to understand areas that need further improvement or those that they are performing well in

**Table 4:** Scenario 3: monitoring thoughts and feelings

Target Group	Children, adolescents and adults
Role of the Robot	Coach or instructor that would engage participants in cognitive restructuring activities
Role of the Participant	Performer
	In this activity, after a clinician has provided psychoeducation regarding thought monitoring, and cognitive restructuring, the social robot could be used by a participant to support these activities outside of the sessions. That is, through a structured conversation with the social robot, the participant could reflect on challenging social situations that happened during the day and identify aspects associated with the situation (e.g., details on the thoughts, feelings, behaviour). The robot could solicit participants’ views on connections between these aspects and provide encouragement throughout. For those participants who did not experience a social situation (due to isolation), the robot and participant could role-play an envisioned social situation during which the participant could be asked to imagine what they might be thinking/feeling and how they might respond. In this way, possible biased ways of thinking could be elucidated in the absence of social activity. Following, the robot could ask questions that prompt the participant to evaluate the certainty of such thoughts, possibility of alternate, balanced, thoughts, and corresponding feelings with the balanced thought. Transcripts of the interactions could be recorded such that the clinician and participant could review them during the session to identify themes as well as challenges with the task
Place/setting	Home, school or clinic
Level of difficulty/variations	As participants are more able to engage in identifying and modifying thoughts, the degree of social robot guidance would be minimized (e.g., fewer number of questions). As well, during role-plays, the robot could be programmed to cue the participant to use previously generated balanced thoughts or coping statements
Potential benefits	Homework assignments, such as tracking thoughts are common within CBT. A social robot could support and guide clients outside of the therapeutic context. Moreover, for individuals who are socially isolated, there may be limited opportunities to engage with others, so creating additional interactions with a social robot may be advantageous as a starting point for practising cognitive restructuring activities

**Table 5:** Scenario 4: practising mindfulness

Target group	Adolescents and adults
Role of the robot	Coach or instructor that would model different mindfulness strategies for the participant
Role of the participant	Learner
	This scenario would begin with a general introduction to stress and social anxiety management. During the psychoeducation stage of this scenario, the participant could learn about different mindfulness exercises, such as regulated breathing. After the psychoeducation stage, the social robot and the participant would engage in a game. In the game, the participant would select a card that lists one of the common physiological symptoms experienced by people with social anxiety disorder. The robot would suggest and model a mindfulness exercise based on the selected card, and then the robot and the participant could practise that exercise. In order to track the effectiveness of the exercises, robots could provide feedback on a client’s physiological signs using sensory data (e.g., Galvanic Skin Response (GSR), Electrocardiogram (ECG), body temperature, etc.) available from wearable sensors
Place/setting	Home, school, clinic or work environments
Level of difficulty/variations	This activity could be performed in a group setting, where all the participants take turns to select a card, and then the robot would model the mindfulness exercises. This variation would allow the participants to practise how to use different mindfulness exercises around other people. Further, the mindfulness exercises could be tailored to specific social situations. For example, the robot could teach the participant about mindfulness exercises that are most suitable when presenting in front of an audience
Potential benefits	With a social robot, participants would learn how to manage cognitions and physiological symptoms associated with anxiety through mindfulness exercises in an interactive manner. With the card game, they could practise different mindfulness exercises in a flexible and fun way. They would also have the opportunity to practise the mindfulness exercises in a repetitive manner. As a companion, the social robot could assist the participant with mindfulness exercises during engagement in feared social situations outside the clinical setting

### Robot Technology: Present and Future

To date, most robots used in therapeutic scenarios in the literature involved some level of remote or Wizard-of-Oz control [165, 168, 176, 255], particularly in the context where the robot is a mediator, i.e., interacts with the client and therapist/carer/teacher or parent - a person who knows best of how the robot, in any specific situation, should respond to the individual person. The need for adaptation and personalization of robot behaviour to individuals has been recognized as an important requirement for robot-assisted therapy and it has been suggested that such scenarios might benefit from some level of robot autonomy [256, 257], as well as suitable interfaces for carers/therapists [233]. If some, or many, behaviours of the robot could be autonomous, this might reduce the cognitive and workload of the clinicians involved, so that they, themselves, could focus on the interventions with the clients rather than being distracted by technical details on how to control the robot. As stated in Sect. 1.5, we do not suggest replacing human experts (clinicians, counsellors, etc) but propose to complement their work using robots as their tools, similar to other digital devices.

Projecting into the future of how robots in therapy could be used in clinical practice, there is a potential for advanced technical robot development, including the ability of a robot to learn and adapt to the individual’s needs and the interaction context to provide personalized behaviours for specific interactions and therapies (e.g., [258–262]). Note, these issues will not be applicable in practice in the near future (at least not in the ‘wild’, i.e., in real settings and without the repeated involvement and monitoring of researchers), but it is an avenue for future research. Such robot adaption to users and their needs could also help sustain long-term interactions in robot-assisted therapy where those needs change, e.g., in situations where such systems are being used over a long time period of months or even years.

Future applications might also benefit from a robot’s abilities to acquire and analyze data during therapy sessions, although this will have to address significant issues of privacy and confidentiality of data in daily practice. Another avenue for future development of robots to help individuals with social anxiety disorders is the tracking of emotional and physiological states with specialized sensors (e.g., some of the widely available devices such as fitbits, Polar sensors etc.) and data that can improve the robot’s social response to emotional and attentional states of the client and may allow the robot to intelligently select psychological interventions to provide the proper interaction for the intended therapeutic goal (e.g., [262–266]). Note, while this is still a very active area of research in the field of robot-assisted therapy, and solutions are not yet ready to be applied in daily practice, if successful in future, algorithms might be able to assess clients’ behaviour and predict their reactions which could yield some degree of autonomy in the interaction of the robot with clients. Also, either manually by the clinician, or with some level of automation, the robot’s behaviour could be adjusted and modified by the therapist to allow changes in the robot’s behaviour over multiple sessions, if required. It needs to be seen in future research, but possibly machine learning techniques might in future allow the robot to adapt autonomously to the changing needs of each client over time and perform certain given tasks such as playing games autonomously, but this is still ongoing and active research. While we insist that it is important that the whole therapeutic process is being led and supervised by therapists, to ensure the appropriateness and achievement of therapeutic goals, in the long term, if successful, interactive robots with some level of autonomy could potentially reduce the burden on therapists [257, 267].

## Scenarios—Integrating Social Robots in Conventional Therapies for Social Anxiety Disorder

In this section, we provide examples of the possible applications of social robots in conventional interventions for social anxiety through four different scenarios. Each scenario has been designed with the aim to illustrate how social robots as complementary tools might be integrated into each conventional therapy available for social anxiety. These scenarios have been proposed to inspire future empirical research; therefore, thorough analyses are required to identify both possible outcomes as well as challenges associated with the evaluation of these scenarios.

The structure of presenting the outlined scenarios is inspired by Robins et al., who developed robot-assisted play scenarios for children with ASD [170, 268]. Each scenario described below targets children, adolescents, or adults because the problems associated with social anxiety disorder are prevalent in all these populations, and all these groups may benefit from innovative methods of intervention [5]. The scenarios also incorporate the aforementioned behavioural and cognitive interventions and different roles of social robots to facilitate the achievement of relevant therapeutic outcomes for individuals with social anxiety disorder. The proposed scenarios are focused on promoting improvement in the emotional symptoms, physiological symptoms, and behavioural manifestations associated with social anxiety disorder. Furthermore, each scenario suggests the place/setting for the therapeutic activity, the variation in the level of difficulty to accommodate different age groups and the benefits the incorporation of social robots would provide to the participants. The duration and frequency of activity would depend on the nature of the clients’ presenting issues and would need to be adjusted based on empirical work in this area. For some reference, cognitive behavioural treatments typically last for 12-16 weeks that include 60-90 minute long sessions [269, 270]. Studies including individuals with social anxiety disorder found that clients needed to undergo 6 to 12 weeks of CBT in order to show any improvement [45, 270].

### Scenario 1: Game of Social Skills

The goal of this scenario is to teach effective use of verbal and non-verbal communication and social skills to children and adolescents with social anxiety, as well as support interaction between peers. Training sessions could involve practising a wide range of social and communication skills while simultaneously allowing individuals to work according to their own abilities in preparation for real-life scenarios. The robot would play the role of a friendly playmate during one-to-one sessions and a mediator in group sessions. Table 2 describes this scenario.

### Scenario 2: Simulating Social Situations

This scenario is proposed to provide exposure to various social scenarios (e.g., conflict resolution, starting conversation, small talk, public speaking, job interview, etc.) and help participants develop comfort and appropriate communication and interaction skills within these contexts. Both the robot and the participant would engage in a role-play [36, 38], where the robot would act as a coach/instructor in this scenario and offer an interactive rehearsal of such social situations to improve participants’ skills and confidence in similar social situations. Table 3 describes this scenario.

### Scenario 3: Monitoring Thoughts and Feelings

This scenario aims to help participants with identifying and modifying maladaptive or biased thoughts about feared social situations. In this scenario, the robot would act as a coach/instructor to support and guide clients outside of the therapeutic context. Table 4 describes this scenario.

### Scenario 4: Practising Mindfulness

In this scenario, participants would learn to manage anxiety-provoking physiological symptoms (e.g., increased heart rate). The robot would act as a coach/instructor in this scenario, and would teach different mindfulness exercises. Table 5 describes this scenario.

### Risks and Concerns for Social Robots in Clinical Practice

Despite their promising nature, we need to acknowledge several concerns surrounding the implementation of social robots in psychological practice. Here we mention three crucial issues.

First, social robots lack human-level perception and decision-making skills, which are critical for sensitive practices, such as diagnosis and intervention. Both diagnosis and intervention entail systematically and sensitively collecting and integrating information about the individuals’ history, social and cultural environments, and verbal and non-verbal behaviours. Social robots can be beneficial for gathering structured information; however, their utility may be reduced during unstructured sessions given their limited ability to learn in real-time.

The second concern regarding social robots is related to the nature of the relationship and dependency that can develop between the social robot and the individual undergoing therapy [271]. While social robots might skillfully mimic emotions and affective responses using e.g., facial expressions, speech, body movements and gestures, they lack genuine human-like emotions and empathy [272]. Thus, long-term dependence on social robots may make it even more challenging for individuals to interact with other people, especially for highly vulnerable individuals. For example, in the context of social anxiety, the perceived discrepancy between the familiar social situation (interaction with robots) and the unfamiliar one (interaction with humans) may cause even greater anxiety. Forming relationships with and dependency on social robots raises serious ethical concerns. As Turkle ([272, p. 514]) put it, “is there a chance that human relationships will just seem too hard?”. We have to remember that robots are not people, they are complex mechanical devices. “With robots, people are acting out ‘both halves’ of complex relationships, projecting the robot’s side as well as their own. Of course, we can also behave this way when interacting with people who refuse to engage with us, but people are at least capable of reciprocation” [272, pp. 504–505]). Thus, for some individuals, relationships with robots might be appreciated because it is ‘easy’, while relationships with humans appear complicated and taxing. Nevertheless, human-robot relationships are *real*, so in the use of social robots in clinical practice one has to avoid presenting technological solutions (robots or otherwise) as substitutes for human-human interaction and relationships. In fact, socially interactive robots could even emphasize this point during the interaction with individuals, e.g., by stating: “I’m here to help you, but I don’t understand human emotions very well, I’m just a robot”.

Third, using technology such as social robots raises concerns about psychological privacy due to disclosure of sensitive or intimate information, as well as data privacy and storage given the lack of transparency of complex technologies and data-driven algorithms, and the increasing reliance on cloud-based data processing [273]. Data privacy concerns are further exacerbated by the nonexistent legal landscape around the development and use of social robots [274]. Due to the aforementioned concerns, robust practitioner supervision and ethical guidelines for the development and use of social robots are necessary before social robots can be widely adopted in psychological practices.

## Conclusion

In this article, we proposed social robots as tools to provide support to both children and adults with social anxiety disorders, with the aim to reduce barriers to treatment utilization and enhance effectiveness. Social robots are embodied agents that can interact in a non-judgmental, flexible, predictable, and engaging manner. In comparison to other forms of technological interventions such as mobile health applications or virtual agents, these characteristics may offer several advantages to integrating social robots in conventional interventions for social anxiety. For example, the social robot’s physical presence could create a rich interactive environment for individuals with social anxiety disorders, which may promote better engagement in activities associated with interventions. Furthermore, situations like COVID-19, which has presented new challenges such as maintaining a physical distance from others, may cause interruptions in delivering mental health care and particularly treatments such as exposure therapy [275]. Therefore, although this has to be shown empirically, the physical presence of social robots may fill such gaps, to some extent, and help reduce the negative impact of these situations.

In addition, incorporation of social robots in conventional interventions has the potential of functioning as a “stepping stone” for individuals with social anxiety, i.e., they could practise social skills and behaviours with a robot before applying their skills to human-human interaction. Engaging in social behaviours without the pressures of interaction with another person can add a novel (likely introductory) layer to treatments. Particularly, individuals with social anxiety who experience excessive fear of negative evaluations may have less tension and stress when they are interacting with a social robot, and the anxiety symptoms experienced during therapy might be more manageable for the individual [31, 186]. This has the potential to make the overall treatment experience more positive for the client. Social robots could also be personalized and adapted to provide the support most suitable for the individual client. However, considering the limited ability to learn and adapt to individual’s needs in presently available, state-of-the-art social robots, significant advances in technology are still needed. Social robots could assume different roles depending on the specific context and user needs. Specifically, social robots could provide clients the benefit of repeated practice and build their confidence through positive feedback. While we have discussed this approach for individuals with social anxiety, certainly, social robots could also be used for individuals who are below the threshold for a diagnosis of social anxiety disorder, but experience some difficulties and distress when participating in social situations.

Incorporating social robots in interventions for social anxiety disorder may not only benefit clients but also clinicians. Social robots can assist clinicians in a variety of therapeutically relevant functions, such as coaching or instructing clients through tasks, providing feedback, assisting with treatment adherence, performing repetitive tasks, and monitoring symptoms and treatment progress. Social robots also offer the advantage of providing interventions in a controlled manner. For example, clinicians, if provided with an easy-to-use interface to program the robot, could control, change, or modify the robots’ behaviours and functions for a specific client and scenario.

Social robots may be particularly useful for aspects of the targeted intervention that include many repetitive steps, which would allow clinicians to focus on the overall process of the treatment and concerns specific to the individual. Social robots can be integrated into therapeutic interventions, taking advantage of the strength of social robots (e.g., providing non-judgmental, reliable engagement) with the strength of clinicians who possess deep expert knowledge, as well as the required understanding of human nature in general, and social anxiety in particular. The latter cannot be provided by social robots. Overall, social robots could offer a new way of delivering interventions. They are not meant to be substitutions for human therapists, rather, social robots should be used as tools to extend and enhance the support provided by clinicians.

To the best of our knowledge, this is the first article proposing the incorporation of social robots in the current conventional CBT approaches for social anxiety. To illustrate the potential applications, we outlined several scenarios and acknowledged possible risks that need to be considered carefully. Note, future research in the application of social robot interventions for social anxiety disorder is needed, and the scenarios we proposed in this article, intended as ‘food for thought’ for the research community and interested researchers, certainly will need to be re-developed and finalized through user-centred and co-design, prototyping, implementation and a series of empirical testing, redevelopment and user feedback, e.g., as it was done in [170]. The goal of this article is to promote exploration and future empirical work and interdisciplinary collaborations to advance the field of robot-assisted mental health interventions in order to benefit clients and clinicians, improving mental health and well-being of those in need.

## Data Availability

Not applicable.
